# Context and Reasons for Bolstering Diversity in Undergraduate Research

**DOI:** 10.3389/fpsyg.2019.00336

**Published:** 2019-02-21

**Authors:** Janelle S. Peifer

**Affiliations:** Department of Psychology, Agnes Scott College, Decatur, GA, United States

**Keywords:** diversity, undergraduate research, publication, inclusion, undergraduate students

## Introduction

The field of psychology has a representation problem, and building diverse undergraduate research labs consistently generate publishable work may play a significant role in the solution. Although no differences exist in key quantitative academic qualifications (i.e., grade point average, graduate record examination scores), Black, American Indian/Alaskan Native, and Hispanic/Latino students of color are less likely to go onto and complete doctoral and other graduate programs in psychology (Callahan et al., 2018). Undergraduate research labs offer a unique opportunity to onboard students of color into the field early in the training-to-workforce pipeline and equip them with the skills to persist, succeed, and, eventually, lead as psychological professionals. These labs scaffold students from various backgrounds as they learn the intricate process of producing quality research for publication. They offer in-depth, ongoing, high-impact mentoring experiences to students that provide the networks necessary to navigate the complexities of graduate school, research, and the professional psychology landscape. Finally, by capturing students' potential during their undergraduate tenure equips them with specialized skills to thrive in both postgraduate academic and professional lives.

The diversification of undergraduate research labs—and the publications that arise from them—helps diversify the field of psychology more generally. Research and lines of inquiry draw from individuals' lenses, identities, and experiences. Henrich et al. (2010) found that while people from Western, educated, industrialized, rich, and democratic (WEIRD) backgrounds account for only 12 percent of the world's population, they represent 80 percent of the samples in published research. Without diversity in research, vital questions go unanswered, key perspectives ignored. Diversity within science can help increase the likelihood of innovation and creativity, as found by Page (2007) exploring the role of diverse perspectives in collective problem solving. Early cultivation of student researchers from diverse backgrounds enriches the breadth and depth of the overall scientific landscape.

## The Current Article

Whereas, another article in this special issue (Ahmad, Sabat, Trump, & King: “Evidence-Based Strategies for Improving Diversity and Inclusion in Undergraduate Research Labs”) delineates evidenced-based action steps, this paper focuses on the context surrounding and reasons for bolstering diversity in undergraduate research. Specifically, the article explores barriers and opportunities to publishing research with individuals from diverse backgrounds and the role that mentored research plays in equity in the field of psychology. This paper hones in on three interconnected facets of diversity—racial, socio-economic, and family educational history—and examines how these facets of identity contribute meaningful breadth to scholarly inquiry. Within the United States social milieu, racial, socio-economic, and family educational history remain deeply linked. While many variations exist, students of color are more likely to hail from backgrounds with less accumulated wealth than White students (American Psychological Association, 2016). These factors affect an undergraduate student's engagement with the research and publication process. This paper will explore considerations for faculty mentoring undergraduate student researchers including historic and current underrepresentation of people of color in the fields of psychological science, inequality in pre-collegiate academic preparation for mentored research, and financial and cultural barriers that may shape students likelihood of participating in publishable research. The conclusion very briefly outlines strategies related to expanding access to high quality mentored research experiences.

## Historical Context

Historically, people of color and people from low-income backgrounds have not only been underrepresented in the field of psychology and in research, but also victimized by it at times. From the Eugenics movement of the nineteenth and twentieth centuries to psychological scientists' promotion of research positing the racial and intellectual inferiority of Black people, psychology's recent history has alienated people of color [see the edited volume by Abramson and Lack (2014), *Psychology Gone Astray: A Selection of Racist & Sexist Literature from Early Psychological Research*]. The makeup of doctoral-level psychologists does not reflect the United States' wider demographic breakdown, with less than 10 percent of the field coming from ethnic/racial minority backgrounds (American Psychological Association, 2015). Publications in our field remain dominated by the voices and perspectives of White, upper class scholars (Henrich et al., 2010). In fact, the vast majority of authors of publications in the top psychological journals are White males from the United States (Murray et al., 2018). This living history presents barriers to undergraduate students from underrepresented backgrounds who cannot see their place in the field and in its research. Faculty members must work actively and invest focused effort to both acknowledge and counteract these variables. By recognizing these complex dynamics at play, current faculty can work in concentrated ways. For example, they can strive to recruit and retain faculty from underrepresented groups, intentionally and thoughtfully recruit students from low income, first generation, and minority backgrounds into research early, and help groom students to produce publishable data through ongoing mentoring and advocacy. The truth is, publications within the field *need* much more inclusivity to stay relevant and respond to some of the most urgent questions of our time (Henrich et al., 2010). Yet, completing publishable research with undergraduate students can prove challenging. The process of recruiting and retaining students from underrepresented groups (i.e., racial minorities, first generation, and low-income students) presents specific complexities and considerations.

## Barriers to Diverse Research

Most faculty ostensibly support the assertion that fostering diversity in psychological research and publication is a worthy aim to increase equity and bolster the quality and breadth of scientific inquiry. Yet, many faculty do not know how (or choose not) to enact inclusive strategies that support students from diverse backgrounds' participation in high quality, publishable research. While some faculty may have an abstract or philosophical commitment to equity, they may balk at or feel stymied by obstacles that may make the enactment of this complex objective challenging. For example, disproportionately, these students hail from school systems that inadequately prepare students to excel in college-level research courses (Engle and Tinto, 2008). First generation students and students from lower socioeconomic backgrounds are more likely to struggle academically (Jury et al., 2017), including in foundational psychology courses. Even students who persist in science majors are less likely than their White peers to participate in cocurricular activities, such as mentored research projects, lab training, or summer research experiences for undergraduates (REUs) that help prepare students to create publishable research (Willis, 2010). Without these vital introductions to active research, students' curriculum vitae do not differentiate them as graduate school applicants and their undergraduate training does not prepare them to thrive if and when they are accepted. Moreover, privileged students and those with parents with higher education backgrounds are more likely to participate in the unpaid, extensive work necessary to produce publishable research as an undergraduate student. First generation and low income students have higher incidences of engaging in part and full time work during their undergraduate tenure and often do so to support the cost of their education (Walpole, 2003; Aronson, 2008).

## Some Strategies for Diversification

Those seeking to increase diversity in publishable research with undergraduate psychology students must seek to understand and actively combat historic and current barriers, differential academic preparation, and financial and social factors. To do so, faculty members may prioritize sharing the full history of psychology with an inclusive lens that acknowledges psychology's background of damaging, racist actions. They can engage actively in discussions that center the long-term implications of this history and explore how it may shape students' relationship with the field, in their classrooms and labs. By foregrounding this reality and raising awareness of the potential dynamics at play, students and faculty have agency to choose how to engage with these challenges in active ways.

Additionally, equity-minded psychology faculty may work to provide additional academic assistance both before students arrive in the major (e.g., academic skill assessments to identify weaknesses, summer, and academic break bootcamps) and during the school year (e.g., tutoring hours, additional academic clinics, additional preparation courses) to help students build the skills necessary to be able to complete publishable research eventually. Offering free tutoring services in courses where students may encounter roadblocks to progression (e.g., Introductory Psychology, lower-level statistics courses) will help students from a variety of backgrounds build a strong analytical foundation. Particularly relevant to publishing, having student tutors with a focus on writing and APA style skills will provide additional support to enable students to develop as scholarly writers. In addition, employing high need, skilled students in work-study or paid tutoring positions can provide a career and graduate school-aligned work experience for students who would otherwise have to work off-campus in non-academic positions that can detract from their study and cocurricular activities. Not only can students benefit from taking advantage of tutoring, but also having students from diverse backgrounds in these roles help build peer mentors who model the research and publication process and early professional networks.

Additionally, faculty labs can seek funding that attracts and enables students who would typically be unable to participate in mentored research to do so. Moreover, folding the process of mentored research into a series of core courses within the psychology major is another way to increase access to all students from all backgrounds to participate in high quality research with support and guidance. Developing several applied research courses for all students scaffolds the full process of research. These courses can demystify the publication process from initial concept all the way through editing and publication. Undoubtedly, publication is a complex, long, and work intensive process that can seem daunting and inaccessible, especially for students from underrepresented backgrounds. The applied nature of the course can center on equipping students with the skills necessary to produce a final, publishable paper. Structurally, the course can fall within an accessible timeframe for students who may not have the flexibility to do summer, after hours, or weekend mentored research.

## Concluding Thoughts

These small, actionable strategies help students create excellent, publishable research rich with the diversity of perspectives of the burgeoning researchers that create it. These adjustments also initiate a cascade of events that help bolster diversity in the future of the field of psychology as a whole. While psychological research still struggles with its living history of inequity, faculty mentors can play a key role as change agents in the field by mentoring students from underrepresented groups with intentionality.

## Author Contributions

The author confirms being the sole contributor of this work and has approved it for publication.

### Conflict of Interest Statement

The author declares that the research was conducted in the absence of any commercial or financial relationships that could be construed as a potential conflict of interest.
